# Recent advances in synthetic methods and applications of silver nanostructures

**DOI:** 10.1186/s11671-018-2450-4

**Published:** 2018-02-18

**Authors:** Zhi Zhang, Wenfei Shen, Jing Xue, Yuanmeng Liu, Yanwei Liu, Peipei Yan, Jixian Liu, Jianguo Tang

**Affiliations:** 1 Institute of Hybrid Materials, National Center of International Research for Hybrid Materials Technology, National Base of International Science and Technology Cooperation, College of Materials Science and Engineering, Qingdao University, Qingdao, 266071 People’s Republic of China; 2 College of Materials Science and Engineering, Qingdao University, Qingdao, 266071 People’s Republic of China

**Keywords:** Silver Nanoparticles, Biosynthetic Methods, Photoelectric, Bio-Sensing, Catalysis, Antibacterial

## Abstract

As the advanced functional materials, silver nanoparticles are potentially useful in various fields such as photoelectric, bio-sensing, catalysis, antibacterial and other fields, which are mainly based on their various properties. However, the properties of silver nanoparticles are usually determined by their size, shape, and surrounding medium, which can be modulated by various synthesis methods. In this review, the fabrication methods for synthesizing silver nanoparticles of different shapes and specific size are illustrated in detail. Besides, the corresponding properties and applications of silver nanoparticles are also discussed in this paper.

## Background

Metallic nanoparticles with the unique optical and electrical properties have been widely investigated during the past decades. Ag nanoparticles (AgNPs) are the most intensively studied metallic nanoparticles because of their unique properties and applications [1–5]. The properties of AgNPs greatly depend on the morphology of particles including the shapes, sizes, and surrounding medium. Large efforts have been devoted to the synthesis methods and the morphological regulation of silver nanoparticles.

Recently, researchers conducted an in-depth study on the excellent function of silver nanoparticles such as photo-electricity [6], catalysis [7], antibacterial [8, 9], biosensors [10], and surface-enhanced Raman scattering (SERS) [11]. So far, AgNPs were successfully prepared by chemical reduction [12–16], photo reduction [17, 18], and laser synthesis [19], etc. However, these methods are usually time and energy consuming. At the same time, they also have the disadvantages of strict preparation conditions and the AgNPs were inhomogeneous in size. Therefore, simple and economical methods, by which the size, shape, and size distribution of AgNPs can be finely controlled, are urgently needed to be developed. Utilizing protective agents is an efficient way to make the AgNPs with good stability and dispersibility. Meanwhile, the agglomeration between particles can be prevented by protective agent. So, protective agents are important to be used for AgNPs synthesis [20].

In this work, the preparation of silver nanoparticles with different shapes such as nanocubes, nanowires, and nanospheres were detailed reviewed. The representative work on preparing silver nanoparticles with different shapes and sizes of 1–10 nm AgNPs, 10–100 nm AgNPs have been reviewed before. As the excellent environmental protection characteristics and simple operation, the new biosynthetic methods to obtain silver nanoparticles which can serve as an alternate to the complex chemical synthetic procedures were singled out as an emphasis. Meanwhile, the properties and applications of AgNPs such as antibacterial, fluorescence, catalysis, and surface plasmon resonance were detailed reviewed as the following. The important application of silver nanoparticles which can be used in nanosensors was highlighted in this review.

This study provides a comprehensive approach which is significant to the investigation of AgNPs. However, it is worth noting that the innovative preparation methods and application breakthroughs still need to be explored.

## Synthetic Methods

Silver nanoparticles were synthesized by various methods, such as seed growth method [21] and stepwise reduction method [22]. Each method has advantages and limitations. So, developing an effective preparation method is still a challenge. Owing to the unique properties and wide applications, the synthesis method of silver nanoparticles is worthy to be optimized. We summarized six types of preparation methods including new biosynthetic methods in this work. We are in the expectation of providing a little help for the workers who are engaging in this field.

### Preparation of Different Types of AgNPs

Recently, researchers were focused on the shape control of AgNPs due to their morphology-dependent properties [23, 24]. Meanwhile, in order to expand their current applications, the preparation of silver nanoparticles with different shapes (such as coral-like shape [25], cage [26], and triangle nanocrystals [27]) aroused a wide range of scientific researches. The formation mechanism and different preparation methods of silver nanoparticles were explored for a long time.

#### Synthesis of Ag Nanocubes

**Fig. 1:**
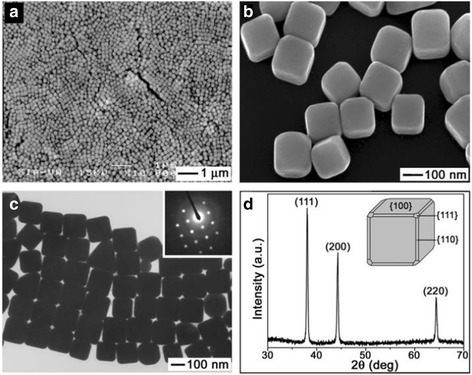
**a** Low- and **b** high-magnification SEM images of slightly truncated silver nanocubes. **c** A TEM image of the same batch of silver nanocubes. **d** An XRD pattern of the same batch of sample, confirming the formation of pure fcc silver [28]

Xia et al. [28–30] massively prepared monodisperse samples of silver nanocubes by reducing silver nitrate with ethylene glycol in the presence of poly vinyl pyrrolidone (PVP). In the synthesis process, PVP was used as protective agents which can stabilize the dispersive silver nanoparticles and prevent the agglomeration. At the same time, the amount of PVP addition can also affect the morphology of AgNPs. Therefore, it is essential to use PVP during the synthesis. It is well known that heating can provide more reacting energy which is beneficial to increase the reducibility of ethylene glycol. In the presence of hydroxyl ions, Ag^+^ was reduced to form silver nanocubes. The advantage of this research is that it can be utilized to prepare homogeneous single-crystal nanocubes. On the nanometer scale, metals that most of them are face-centered cubic (fcc) tend to nucleate and grow into twinned and multiply twinned particles (MTPs) owing to their surfaces bounded by the lowest-energy facets on the nanometer scale [31]. Moreover, this structure is beneficial to be applied in the field of photonics, catalysis, and SERS-based sensing. The picture (Fig. 1) shows the SEM, TEM, and XRD images of silver nanocubes. These silver nanocubes had a mean edge length of 175 nm, with a standard deviation of 13 nm. Their surfaces were smooth, and all corners and edges of these particles were slightly truncated. This structure may be used for drug delivery systems by injecting drug into the truncated corners.

A novel silver nanoparticle was published by Yam et al. [32] that utilized Cetyltrimethyl Ammonium Bromide (CTAB) as surfactant in aqueous solution. The bromine ion can react with the silver ammonia complex ([Ag (NH_3_) _2_]^+^) to produce AgBr precipitation, and silver ions will be released slowly in the subsequent reaction. At the same time, the residual silver ions were reduced by glucose, and a size of ~ 55 nm nanosilver cube was formed with the coated surfactant. The surfactant CTAB can be adsorbed on the surface of AgNPs by physical adsorption. On this account, the agglomeration and scale growth of AgNPs can be effectively controlled by inhibition. Because of the existence of CTAB, it is possible to obtain AgNPs with uniform dispersion and suitable size.

It takes a long time to prepare nanosilver cubes reported by Xia and Yam synthetic’s method. But silver nanoparticles can be rapidly produced by the microwave method. Saraf et al. [33] prepared silver nanocubes by utilizing large quantity of gold seed in the presence of polyelectrolyte and microwave heating for 60–120 s. The experiment indicates that the polyelectrolyte guides the growth of the particle in a specified crystallographic direction resulting in the faceted particle, i.e., a nanocube. At present, the preparation of silver nanoparticles by polyol method is more mature.

#### Synthesis of Ag Nanowires and Nanorods

**Fig. 2:**
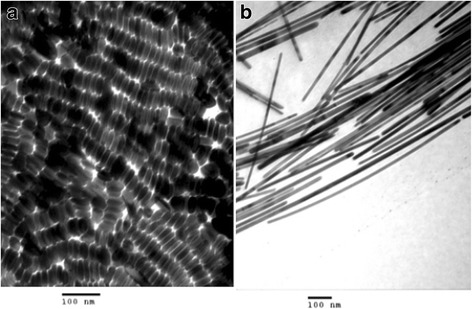
**a** Shape-separated silver nanorods from a preparation with 0.06 mL seed. **b** Shape-separated silver nanowires [34]

Murphy et al. [34] reported that nanorods and nanowires can be prepared successfully by using ascorbic acid to reduce AgNO_3_ in the presence of Ag seed, the micellar template CTAB, and NaOH. The average diameter of Ag seeds is 4 nm. In this work, the concentration of seed and base relative concentration of Ag^+^ play a key role in making larger aspect-ratio nanomaterials. CTAB is also necessary for preparing a high yield of rods. The picture of TEM (Fig. 2) shows the shape of nanorods and nanowires.

Silver nanorods were prepared by Lee et al. [35]. In seed-mediated growth method, small metal particles are prepared first and later used as seeds for the preparation of nanorods. The silver seeds were prepared by reduction of silver ions with sodium borohydride in the presence of sodium citrate dihydrate as stabilizer. These silver seeds were added into the solution containing more silver salt, an ascorbic acid (weak reducing agent), and a CTAB. In this study, the reaction temperature and pH controlled the aspect ratio and uniformity of the resulting rods. Increase of reaction temperature led to decreased aspect ratio of silver nanorods and increased size of the monodispersed particles. Also, increase of pH showed the similar results. When reaction temperature and pH were increased, the reduction-rate of silver was further increased. In experiment, silver nanorods with high aspect ratio and monodispersity were synthesized under the condition of 30 °C and pH 10.56. Silver nanorods were synthesized by electrochemical methods from an aqueous solution of AgNO_3_ in the presence of polyethylene glycol (PEG) by Zhu et al. [36]. It was found that the concentration of AgNO_3_ and PEG affected the formation of the nanorods.

Murphy et al. provided a better method for preparing silver nanowires, but Sun’s [37, 38] synthetic way is more refined. They synthesized silver nanowires by reducing AgNO_3_ with ethylene glycol in the presence of seeds and PVP. The reaction mechanism is as follows:

1 $$ {2\mathrm{H}\mathrm{OCH}}_2\hbox{--} {\mathrm{CH}}_2\mathrm{O}\mathrm{H}\to {2\mathrm{CH}}_3\mathrm{CHO}+{2\mathrm{H}}_2\mathrm{O} $$

2 $$ {2\mathrm{CH}}_3\mathrm{CHO}+{2\mathrm{Ag}\mathrm{NO}}_3\to {\mathrm{CH}}_3\mathrm{CO}\hbox{--} {\mathrm{COCH}}_3+2\mathrm{Ag}+{2\mathrm{HNO}}_3 $$

**Fig. 3:**
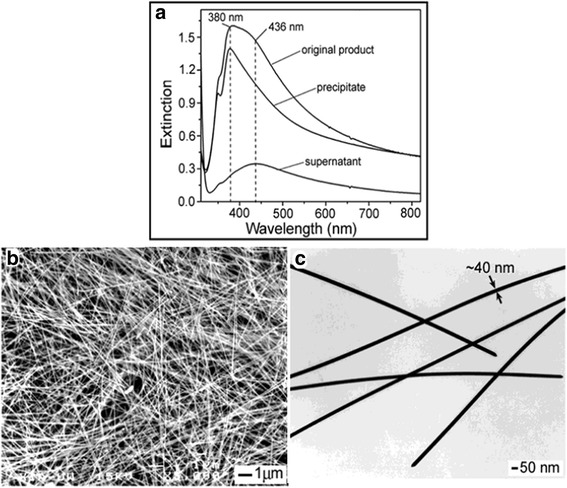
**a** UV-visible extinction spectra of the final product before and after 3 cycles of centrifugation and separation. **b** SEM and **c** TEM images of a purified sample of silver nanowires [37]

Next, AgNO_3_ and PVP were guttatim added into the reaction system, allowing the nucleation and growth of silver and forming uniform shape and size of nanowires. Silver nanowires with diameters of 30–40 nm, and lengths up to ∼ 50 μm were produced by this way. The effects of various reaction conditions (temperature, reacting time, and seeding conditions) on the morphology and size were discussed in this review. Figure 3 shows the shapes and sizes of the purified nanowires.

In the UV-vis spectrum (Fig. 3a), it can be seen that silver nanowires show an obvious blue shift in UV-vis absorption compared with original sample after purification. The UV-vis absorption peak appears at 380 nm. Figure 3c shows the width of these nanowires is 40 nm. The nanowires with the same width can be obtained, which is the best advantage of this work. These nanowires may be used to prepare conductive films [39] and efficient organic solar cells [40], etc.

Through further studying how PVP reacted with silver nanowires, Xie et al. [41–43] concluded that the PVP monolayer reacts with Ag nanowires via Ag–O bonds. On this basis, Xie et al. [44, 45] observed the existence of multiple twins in experiments that proved Xia on multiple twinning is one of the key factors in the formation of Ag nanowires. Controlling the initial amount of silver nitrate or reducing the initial rate of reduction of silver nitrate is conducive to the formation of silver nanowires in the solution [46, 47]. The specific method that they utilized is to control the reaction of the metallic salt and silver nitrate by adding chlorine ions to the reaction solution or reducing the release rate of silver ions.

Tang et al. [48] synthesized size-controlled silver nanowires by adding stainless steel mesh to the system which has higher ion concentration. It is mainly that the stainless steel mesh can react with nitric acid which can be helpful to prevent the corrosion of the multiple crystal grains. In the presence of chloride ions, they prepared uniform silver nanowires by using hydrothermal method, microwave method, and other experimental methods [49, 50]. Silver sulfide nanoparticle is a new type semiconductor which is easily synthesized  through the reaction of sulfur ions with silver ions.  Silver sulfide nanoparticles can provide electrons and make silver ions adsorb on the surface of it and act as a core and reducing agent. At the same time, silver atoms can also be deposited on the surface of Ag_2_S to form Ag_2_S@Ag seeds and play a role of self-catalytic reduction, which is conducive to the formation of silver nanowires [51].

#### Synthesis of Ag Nanospheres

**Fig. 4:**
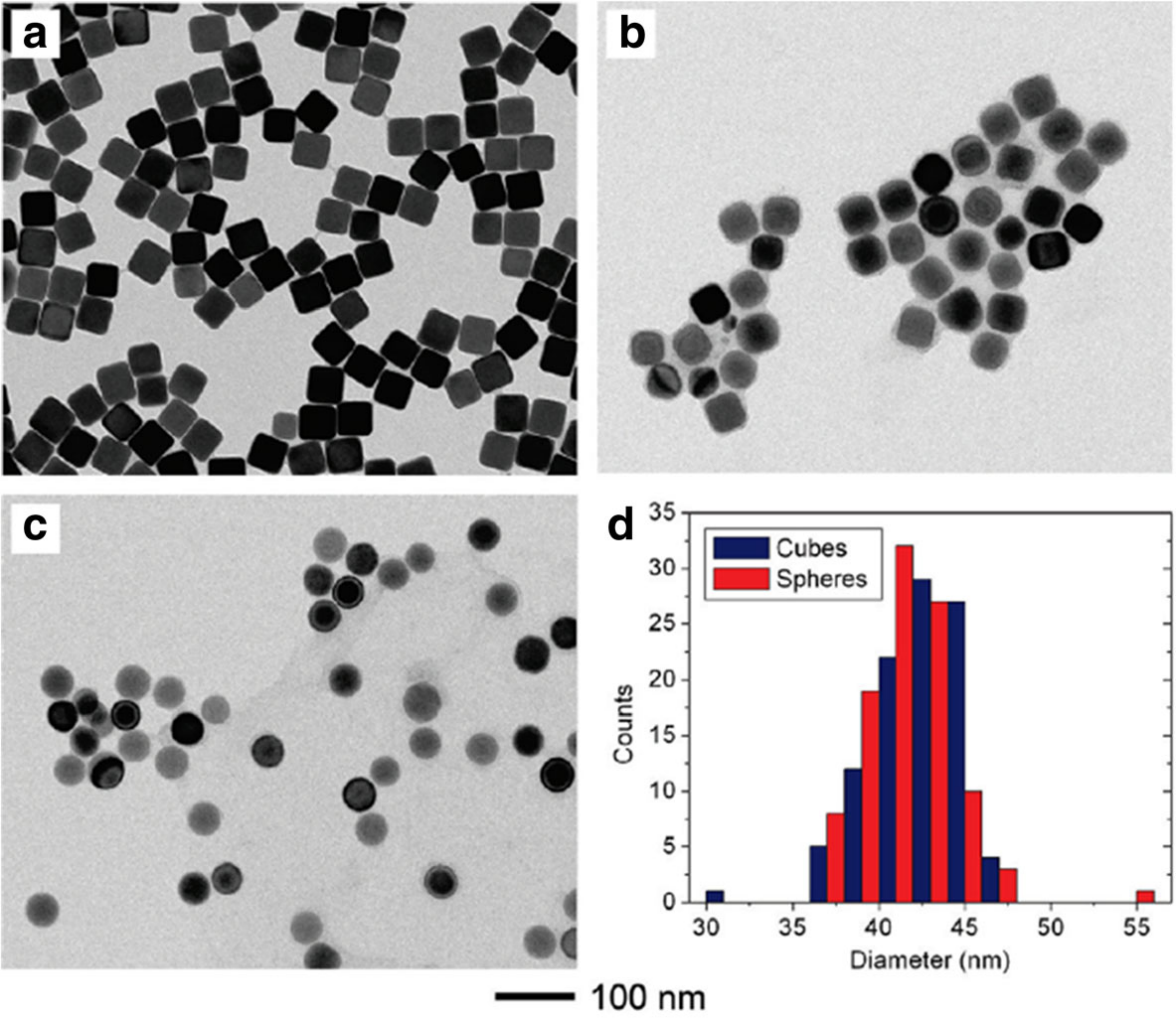
TEM images (**a–c**) of 42 nm Ag cubes capped with PVP when etched with different volumes of 0.5 mM ferric nitrate. **a** 0 μL. **b** 10 μL. **c** 100 μL. **d** Size distributions calculated from 100 particles in parts **a** and **c** [57]

Quasi-spherical silver nanoparticles, which are commonly synthesized by chemical reduction method, are reported by lots of works because silver atoms easily tend to form spherical structure during synthesis process of silver nanoparticles. In the chemical reduction process, commonly employed reduction agents include sodium borohydride [52], sodium citrate [53], hydrazine hydrate [54], ascorbic acid [55], and hydrogen [56]. Of all the research teams, Xia’s team is the most detailed and comprehensive in studying. In order to obtain the high quality of single-crystal Ag nanospheres [57], they utilize a new method based on wet etching which is different from chemical reduction. By rapidly mixing the suspension of uniform Ag nanocubes with a small amount of ferric nitrate or ferricyanide-based etching solution, they could either truncate the sharp corners and edges to form rounded nanocubes or obtain nanospheres without sharp features which have the same diameter as the original cubes. Because previous synthetic methods are unable to prepare uniform spheres larger than ∼ 35 nm. Notably, this method could be used to produce uniform Ag nanospheres with a broad range of sizes and open up new possibilities for fundamental studies on SERS. In this work, the Ag spheres can be prepared with the smallest size of 25 nm and the largest size of 142 nm. Figure 4 shows that the silver nanocubes were etched into quasi-nanospheres.

AgNPs prepared with this method have the regular shape and uniform size. These silver nanospheres with regular shape and uniform size can be used to prepare uniform gold nanocage which can be utilized for biological targeted drug delivery [58].

**Fig. 5:**
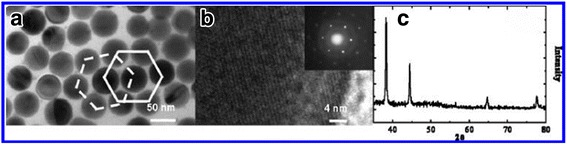
**a** TEM and **b** HRTEM images of the silver nanospheres prepared at 260 °C for 24 h with molar ratio of PVP to AgNO_3_ of 8, and the SAED pattern (inset) of an individual silver nanosphere with a diameter of about 50 nm. **c** Powder XRD pattern taken from the same batch of sample [59]

Liang et al. [59] reported a novel technique for fabricating monodisperse silver nanoparticles. The PEG is utilized both as a solvent and reducing reagent and the PVP is utilized as capping agent for the synthesis of monodisperse silver nanoparticles. To obtain uniform nanospheres with an average diameter of 54 nm, Liang utilized the PVP/AgNO_3_ molar at a ratio of 8 at 260 °C. Figure 5 shows the TEM, HRTEM, and XRD images of silver nanospheres.

It can be seen that the size of Ag nanospheres is uniform from the TEM image. Moreover, the synthesis method is simple and it can be applied to mass production. Of course, there are many other papers on the study of spherical nanosilver which is also worth learning. But in this work, we will not repeat them. Next section, we will describe three types of preparation methods by which different sizes of silver nanoparticles are fabricated. We expect to provide a little help for the workers who are engaging in studying the effect of size and performance.

### Preparation of Different Sizes of AgNPs

It is universally acknowledged that silver nanoparticles with different sizes have a significant influence on the performance of materials. Nevertheless, we find that few papers systematically describe the preparation methods of silver nanoparticles with different sizes. So, we introduced some synthetic methods in the following section with the hope that it can help someone who wants to get definite size.

#### Fabrication of 1–10 nm AgNPs

Small size silver nanoparticles were generally produced through the fast reducing process in which sodium borohydride was employed as reduction agents, and the size and shape of the produced particles were not uniform. Shekhar et al. [60] prepared 5–10 nm silver nanoparticles by mixing different proportions of sodium borohydride and sodium citrate which are used as reductant (using sodium borohydride to preferentially reduce fast nucleation and sodium citrate reduction again to keep steady growth). By this method, the uniform size and shape of AgNPs were obtained. The following Table 1 shows the designed conditions for the synthesis of different-sized silver nanoparticles.

**Table 1:** Designed Conditions for the Synthesis of Different-Sized Silver Nanoparticles^a^ [60]

Particle size	Silver nitrate (mol dm^− 3^)	Sodium borohydride (mol dm^− 3^)	Trisodium citrate (mol dm^− 3^)	Volume of reactants (ml)	pH	Temp. (T^a^-T^b^) (°C)	Yield (%)	Particle conc.(particles per ml)
5	1.00 × 10^−03^	2.00 × 10^− 03^	4.28 × 10^− 03^	x48y2	10.5	60–90	78.2	1.03 × 10^15^
7	1.00 × 10^−03^	2.00 × 10^− 03^	3.55 × 10^− 03^	x48y2	10.5	60–90	67.4	3.31 × 10^14^
10	1.17 × 10^−03^	2.00 × 10^− 03^	2.00 × 10^− 03^	x48y2	10.5	60–90	82.3	1.53 × 10^14^
15	1.00 × 10^−03^	1.00 × 10^− 03^	1.06 × 10^− 03^	x48y2	10.5	60–90	77.9	3.49 × 10^13^
20	1.00 × 10^−03^	1.00 × 10^− 03^	3.55 × 10^− 03^	x48y2	10.5	60–90	~ 84	1.57 × 10^13^
30	4.00 × 10^−03^	1.00 × 10^− 03^	3.55 × 10^− 03^	x45y5	10.5	60–90	70.5	1.71 × 10^13^
50	1.22 × 10^−03^	5.00 × 10^− 03^	2.00 × 10^− 03^	x45y5	10.5	60–90	58.4	9.01 × 10^11^
63	2.00 × 10^−03^	5.00 × 10^−03^	3.54 × 10^− 03^	x40y10	10.5	60–90	~ 61	7.25 × 10^11^
85	2.00 × 10^−03^	5.00 × 10^− 03^	1.77 × 10^− 03^	x45y5	10.5	60–90	68.3	3.43 × 10^11^
100	2.00 × 10^−03^	5.00 × 10^− 03^	1.77 × 10^− 03^	x40y10	10.5	60–90	64.6	1.99 × 10^11^

^a^x-total volume of NaBH_4_ and TSC, y-volume of AgNO_3_ added drop wise to x, T^a^-first stage temperature, T^b^-second stage temperature(°C)

**Fig. 6:**
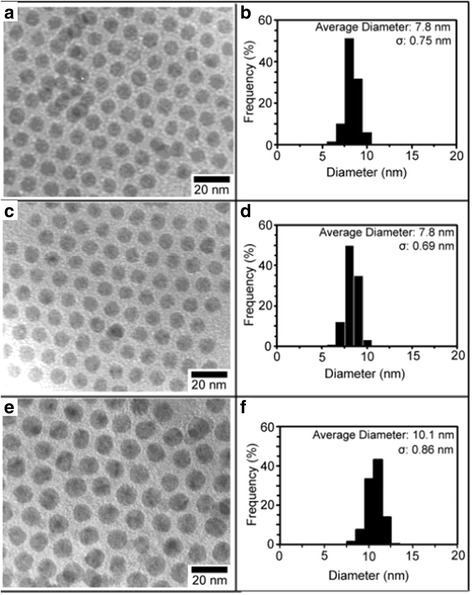
Bright field TEM images and the corresponding particle size distribution analyses of AgNPs obtained at an oleic acid/silver trifluoroacetate molar ratio of 10:1 for durations of (**a**, **b**) 30, (**c**, **d**) 90, and (**e**, **f**) 150 min [61]

Lin et al. [61] prepared 7–10 nm silver particles which are uniform in shape and size in 2003. A simple synthetic method was described that they prefer to directly prepare narrowly dispersed silver nanoparticles rather than use size-selection processes by thermal reduction of silver trifluoroacetate in isoamyl ether in the presence of oleic acid. This direct synthesis is synthetically easy to control and able to obtain AgNPs with diameters in the range of 7–10 nm and narrow size distribution. Instead of using the traditional approach which involves the precursor of silver salts and reducing agent in a solvent, a single-source precursor in an organic solvent was used in the experiment. For this reason, they chose silver trifluoroacetate as single-source precursor because it is readily available and can be thermally reduced to silver metal at various temperatures. Last, they transformed the diameter of AgNPs by adjusting the molar ratio of the oleic acid to silver trifluoroacetate. The following Fig. 6 shows the bright field TEM images and the corresponding particle size distribution analysis of AgNPs which were obtained at an oleic acid/silver trifluoroacetate molar ratio of 10:1 for durations of (A, B) 30, (C, D) 90, and (E, F) 150 min.

**Fig. 7:**
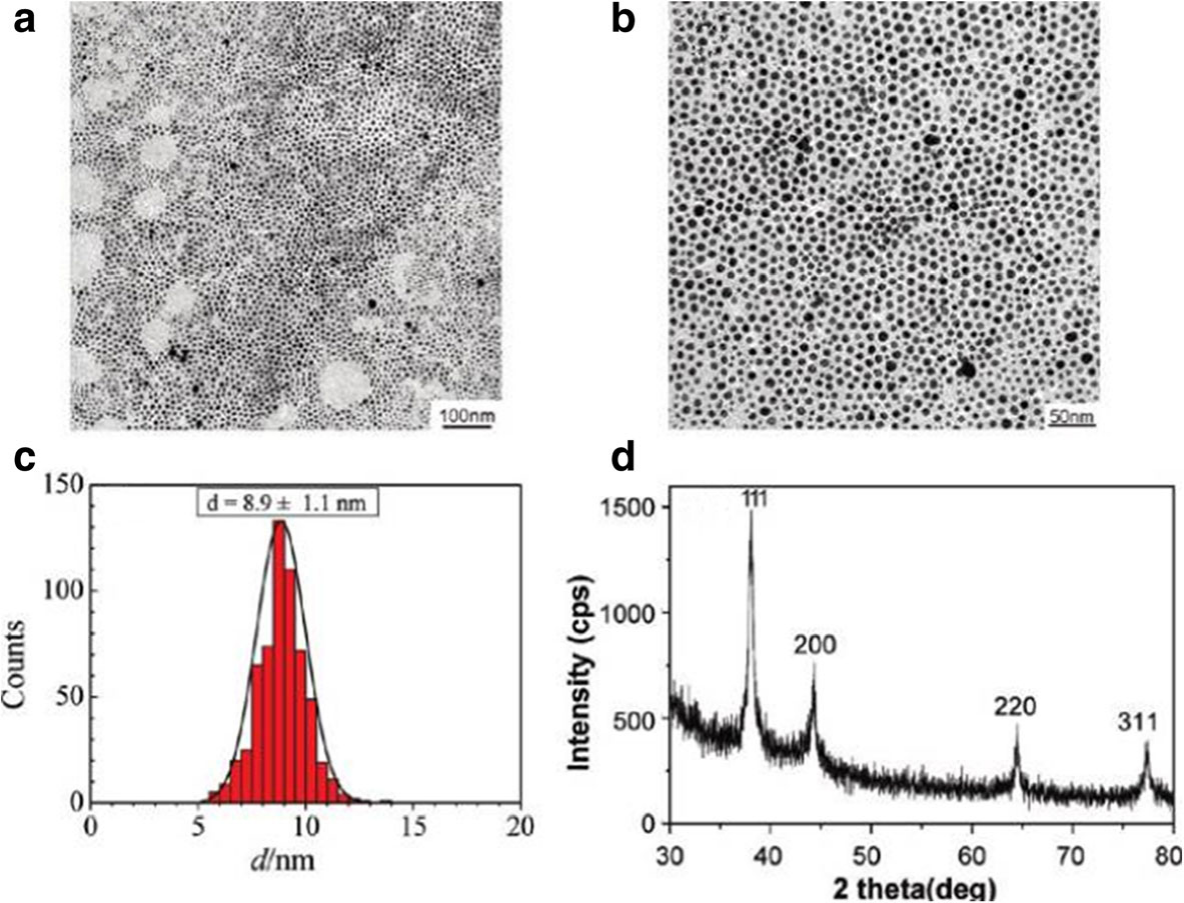
**a**, **b** TEM images at two magnifications of silver nanoparticles collected from the reaction system after adding NaOH at 90 C for 1 h. **c** Corresponding histogram of the silver nanoparticles size distribution. **d** XRD pattern of silver nanoparticles [62]

A simple way to synthesize monodisperse silver nanoparticles with diameter less than 10 nm at high concentration was found by Yang et al. [62]. They pioneered a method that aniline was used as a reducing agent and dodecyl benzene sulfonic acid (DBSA) as a stabilizer. Upon the addition of excess NaOH to the DBSA aniline AgNO_3_ system, the formation of silver nanoparticles was almost complete in just 2 min at 90 °C (with 94% yield). Moreover, the average size of those resultant silver nanoparticles is 8.9 ± 1.1 nm, and the colloid can be stored for more than 1 year at ambient temperature. The Fig. 7 is the TEM, DLS, and XRD images of AgNPs.

**Fig. 8:**
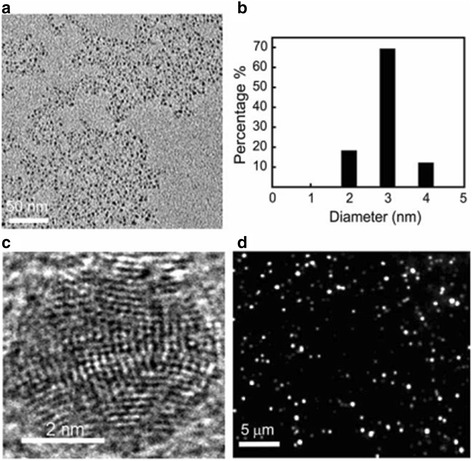
Size distribution, structure, and luminescence emission of the 3 nm silver nanoparticles created using solid-phase thermolysis. **a** Low-resolution TEM image of these nanoparticles. **b** Size distribution of the nanoparticles determined from TEM. **c** High-resolution TEM image of such a small silver nanoparticles shows a highly multi-domain structure. **d** Luminescence image of these small silver nanoparticles taken under 488 nm laser excitation at ~ 10 W/cm^2^ [63]

The methods for synthesizing small size silver nanoparticles described above are all in the liquid phase system. However, Zheng et al. [63] synthesized silver nanoparticles with the diameter of 2–4 nm in the solid-phase system. They synthesized luminescent and Raman active silver nanoparticles by taking advantage of thermal reduction method. Figure 8 shows the size distribution, structure, and luminescence emission of 3 nm silver nanoparticles which are created by solid-phase thermolysis.

#### Fabrication of 10–100 nm AgNPs

**Fig. 9:**
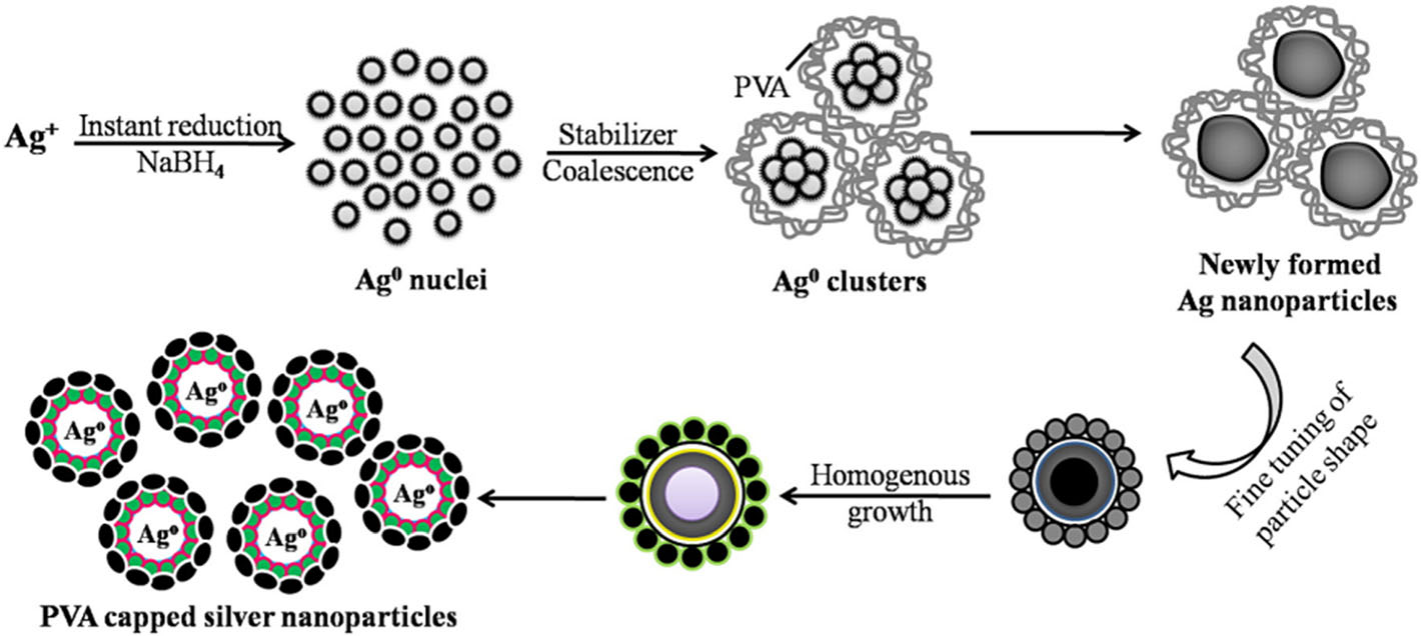
Schematic representation of size-controlled AgNPs synthesis employing the chemical reduction method [67]

By irradiation with 6 MeV electrons, AgNPs with the diameter of 10–60 nm were synthesized by Bogle et al. [64] in the mixture of silver nitrate and PVP. This method has many advantages such as preparation efficiency, high productivity, and few by-products. Abid et al. [65] prepared silver nanoparticles by using the laser irradiation similar to the above work. The difference is that they utilized sodium dodecyl sulfate (SDS) as capping agent to mix with silver nitrate and silver nanoparticles with size of 13–16 nm can be prepared. The particle size is controlled by laser intensity and the initial concentration of SDS surfactant. With the use of ascorbic acid reduction, spherical silver particles with size of 30–72 nm were synthesized by Qin et al. [66]. Meanwhile, the size of silver nanoparticles decreased as pH of the reaction system increased from 6.0 to 10.5. Ajitha et al. [67] utilized chemical reduction by adjusting PH to obtain 14–31 nm AgNPs. They used ethanol as a solvent, sodium borohydride as reducing agent, and poly-vinyl alcohol (PVA) as capping agent. Figure 9 shows the formation mechanism of these silver nanoparticles.

**Fig. 10:**
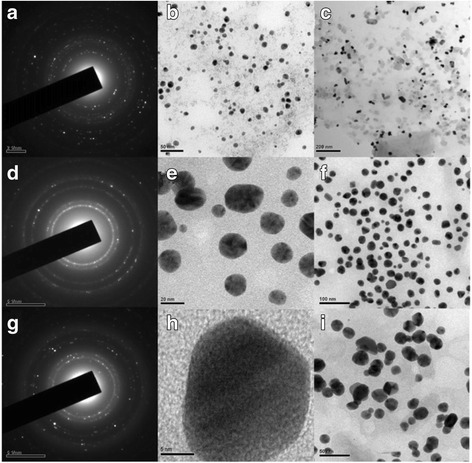
TEM images and SAED patterns of colloids the volume of leaf extract 4 ml (Ct4) (**a**–**c**), Ct3 (**d**–**f**), and Ct1 (**g**–**i**) nanoparticles [72]

Similarly, Ag particles with the diameter of 15–21 nm were synthesized by Silvert, P. Y. et al. [68] who utilized an ethylene glycol–PVP solution to reduce silver nitrate under specific temperature conditions. The uniform quasi-nanospheres were synthesized by this method. In order to detect the solubility of silver nanoparticles with different sizes, 10–80 nm Ag particles were prepared by Rui Ma et al. [69]. They prepared dispersive colloidal silver by the polyol process which is a mature preparation method [70]. Their methods of preparation are based on Silvert, P. Y, by changing the type of protective agent. Recently, the green synthesis study is very fiery and researchers usually utilize amino acid or peel reduction Ag^+^ to synthesize silver nanoparticles. Because an environmentally friendly synthetic method can overcome the problem that is the production of toxic substances in physical and chemical preparation methods. Among them, Maddinedi et al. [71] utilized tyrosine as reducing and capping agents to prepare 13–33 nm silver particles by adjusting PH from 12 to 10. Mandal et al. [72] obtained the same results. They used the leaf extract of Cinnamomum tsoi as reducing and capping agents to prepare 11–31 nm silver particles by adjusting the intrant volume of leaf extract of Cinnamomum tsoi. Figure 10 shows the TEM and SAED patterns of AgNPs.

**Fig. 11:**
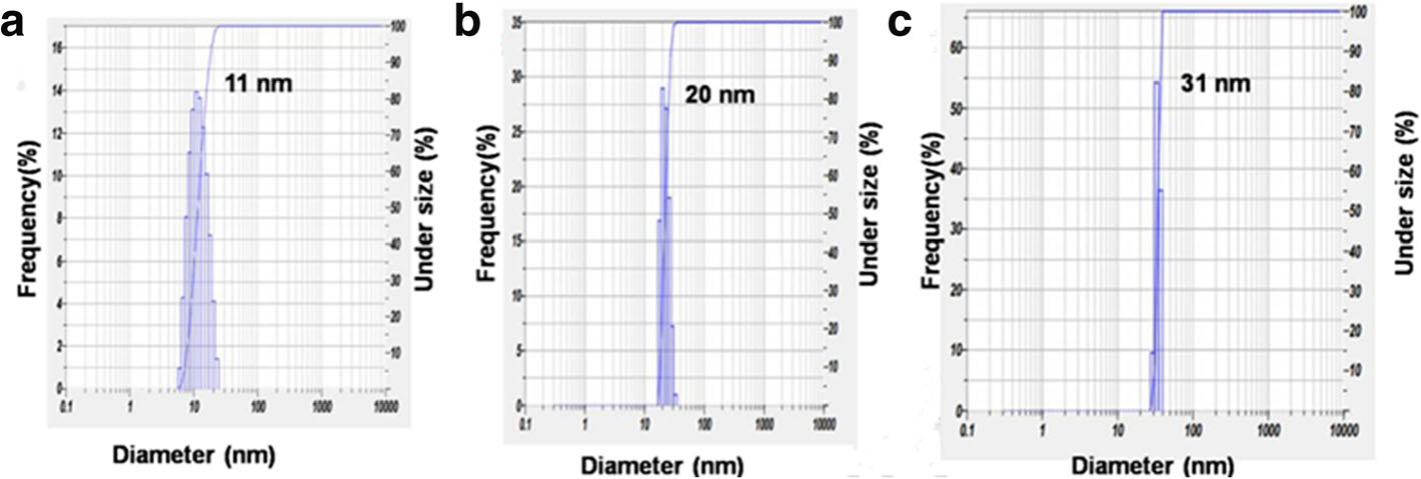
Average particle sizes obtained for AgNPs Ct1 (**a**), Ct3 (**b**), and Ct4 (**c**) [72]

Figure 11 shows the dynamic light scattering (DLS) of AgNPs that the volume of leaf extract was varied as1, 3 and 4 ml.

Of course, there are many other methods to prepare 1–100 nm silver particles. The above papers are only typical. We do this work because we hope to help someone who wants to synthesize definite size. In conclusion, the preparation of silver nanoparticles should be guided towards friendly synthesis and controllable size.

### Preparation of AgNPs by Biosynthetic Methods

The biosynthesis of metallic nanoparticles using biological systems has evolved to become an important area of nanobiotechnology. Biosynthetic methods are better candidates for preparing AsNPs due to enviromental friendly techniques are adopted in their fabrication process and the products are suit for bioapplications.  Herein, biosynthetic methods have a prospect for development and research. So, we have a detailed discussion for some synthetic cases. In 1999, Klaus et al. [73] firstly used *Pseudomonas stutzeri* to synthesize silver nanocrystals with the size of 200 nm. Subsequently, the use of other strains to prepare silver nanoparticles has been developed greatly, such as the aspergillus flavus and trichoderma. And Kazemi et al. [74] successfully synthesized Ag nanoparticles by utilizing Geotricum sp. Geotricum sp. was grown in Sabro Dextrose Agar (SDA) medium at 25 ± 1 °C for 96 h. The mycelia is used to convert silver nitrate solution into nanosilver. Silver nanoparticles were extracellularly synthesized using these fungi (Geotricum sp.). This efficient, eco-friendly and simple synthetic method can be used to synthesize Ag nanoparticles of 30–50 nm. Due to the use of the room temperature conditions and the absence of harmful reducing agents, we can regard this method as environment friendly and low cost. Recently, laryssa et al. [75] prepared silver nanoparticles by utilizing the cell-free filtrate of nematophagous fungus Duddingtonia flagrans. In this study, they reported a simple biological process for the synthesis of AgNPs using the nematophagous fungus D. flagrans. Compared with biosynthesis which is a cheap, environment friendly and high-yield process, extracellular synthesis which does not need the additional treatment to separate particles from living cells is a more simple process. Biosynthesized and functionalized AgNPs have good stability and high yield, and the excellent properties of antibacterial, antifungal, antiviral and anticancer make them have a promising future in the therapeutic applications, which potentiates new experimental designs on using the fungus D. flagrans.

It can be seen that the type of biological micro-organism will be the latest research direction in nanosilver study.

## Properties and Applications of AgNPs

### Properties and Applications of AgNPs on Antibacterial

In recent years, antibacterial properties of Ag nanomaterials gradually aroused people’s concern and a lot of antibacterial applications were reported [76, 77]. The antibacterial AgNPs with different shapes were researched by Helmlinger et al. [78]. By studying the cytotoxicity and antibacterial effect of four types silver nanometals, it can be seen that silver nanoparticles with different shapes own equal cytotoxicity, but it has different antibacterial effect. Meanwhile, particles with a higher specific surface area are more toxic for bacteria than particles with smaller specific surface areas. The dissolution kinetics is correlated to the estimated specific surface area of the particles where particles with a higher specific surface area dissolve faster than particles with a smaller one. The difference in the dissolution rate may be exploited to synthesize silver nanoparticles with a relative higher antibacterial effect and a lower cytotoxic effect towards tissue. However, Helmlinger et al. did not give a further detail study on the antibacterial effect of different sizes of AgNPs.

**Fig. 12:**
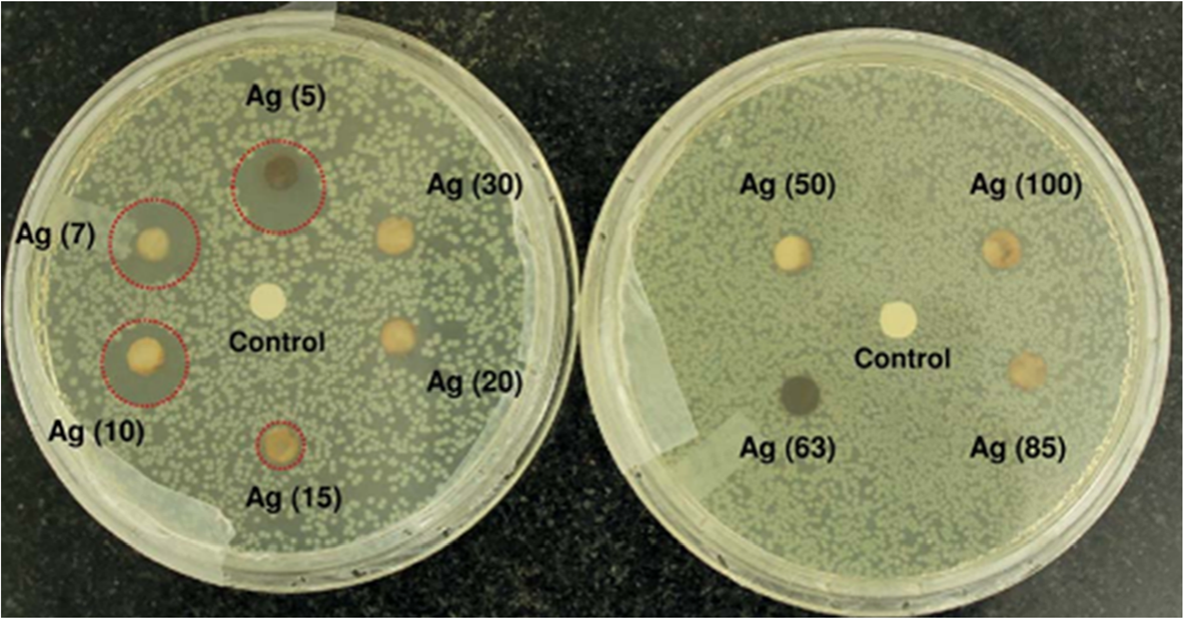
Disk diffusion tests for different-sized silver nanoparticles against the *E. coli* MTCC 443 strain. The zone of inhibition is highlighted with a dashed circle indicating a noticeable antibacterial effect [60]

The antibacterial properties of silver particles with different sizes were studied by Agnihotri et al. [60]. It can be seen that 5 nm nanoparticles have the best antibacterial properties. It was found that the smaller particles exhibited the better antibacterial properties. The Fig. 12 shows the antibacterial properties of the different-sized silver nanoparticles.

Silver extends its antibacterial properties by combining with other materials. Research about combining with other materials included SiO_2_@Ag [79], PLLA microcapsules combined with silver nanoparticles [80], electrodeposited chrome/silver nanoparticles (Cr/AgNPs) [81], graphene quantum dot/silver nanoparticles [82], Ag-decorated polymeric micelles with curcumin [83] and so on.

**Fig. 13:**
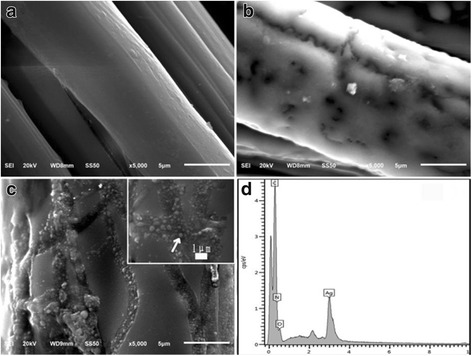
Surface morphologies of pristine silk fiber (**a**), (PAA/PDDA)8 film-coated silk fiber (**b**), and AgNPs-(PAA/PDDA)8 film-coated silk fiber (**c**). Inset: SEM image with higher magnification. (**d**) EDS spectrum of AgNPs-(PAA/PDDA)8 film-coated silk. The arrow indicates the point randomly selected for the EDS analysis [85]

All the above studies are about the antibacterial properties of AgNPs. Next, we introduced the silver nanoparticles for antimicrobial application. It was found that the silver nanoparticles can be directly utilized as antibacterial agents which have been also testified by Kujda et al. [84]. It is shown that silver particles attach to the bacteria surface inducing disintegration, which enables their penetration inside the bacteria. In the future, the antibacterial properties of silver nanoparticles should be applied in industry by combining with other materials. For example, Meng et al. [85] made silver nanoparticles adhered to multilayered film-coated silk fibers with the aim to get antibacterial application. The as-prepared silk could effectively kill the existing bacteria and inhibit the bacterial growth, demonstrating the antimicrobial activity. Moreover, the release of Ag^+^ for the modified silk can last for 120 h, rendering the modified silk sustainable antimicrobial activity. This work may provide a novel method to prepare AgNPs-functionalized antimicrobial silk for potential applications in textile industry. Figure 13 shows the surface morphologies of pristine silk fiber and coated morphologies of silk. By the EDS analysis, we can make sure that nanosilver was coated with silk.

**Fig. 14:**
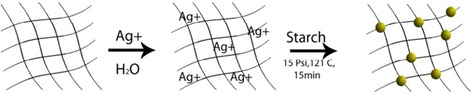
Schematic diagram of impregnation of silver nanoparticles on cotton fabric [86]

Other people like Zulfiqar Ali Raza et al. [86] investigated single-bath fabrication and impregnation of silver nanoparticles on enzymatic pretreated cotton fabric by using starch both as reducing as well as stabilizing agent under the autoclave conditions of 103.42 kPa, 121 °C for 15 min. The silver nanoparticles impregnated cotton fabrics showed good durable antibacterial activity against *Escherichia coli* and *Staphylococcus aureus* strains. Figure 14 shows the formation mechanism of impregnation of silver nanoparticles on cotton fabric.

Recently, silver nanoparticles were coated with zirconia by Yamada et al. [87] for antibacterial prosthesis. In view of the pronounced antimicrobial properties and small toxicity of AgNPs, the biocompatible AgNPs-coated yttria-stabilized zirconia can be potentially utilized to control dental caries and periodontal disease. Maybe the inspiration about wound repair will be obtained by this study. The excellent antibacterial properties of silver nanoparticles can be revealed by the above studies. Moreover, this work will help someone who wants to do further research on antibacterial.

### Properties and Applications of AgNPs on Fluorescence

**Fig. 15:**
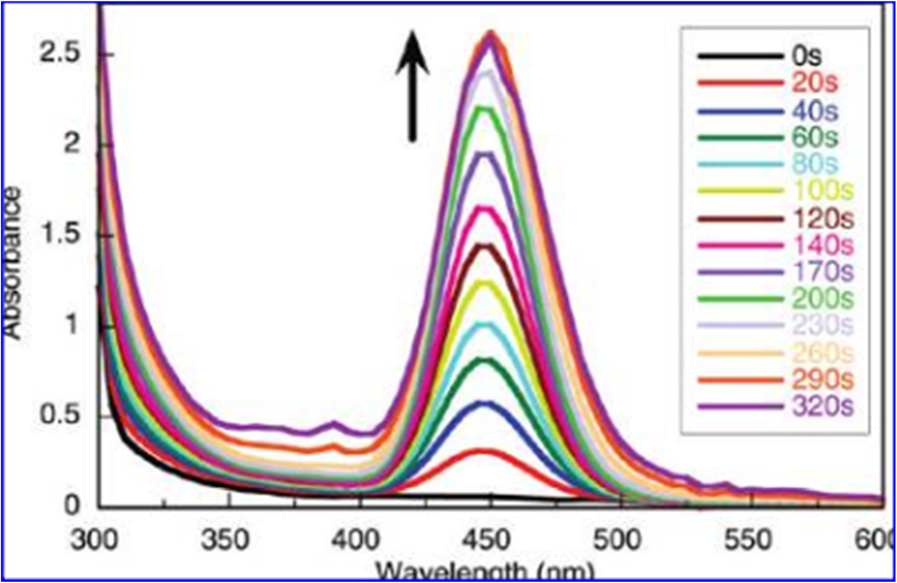
UV-vis absorption spectra following irradiation (350 nm, four lamps) of a toluene solution containing 2 mM silver trifluoroacetate, 2 mM I-2959, 2 mM cyclohexylamine. Reaction performed and monitored directly in a 0.7 × 0.3 cm quartz cuvette [92]

**Fig. 16:**
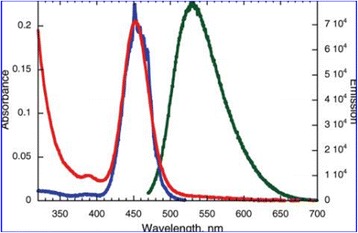
Absorption (red), emission (green), and excitation (blue) spectra of Ag particles after 4 min of irradiation in THF under the conditions of Fig. 15 and resuspension in toluene [92]

Because nanomaterials with fluorescent property have a great application prospect. Many efforts have been devoted to study the fluorescent property [88, 89]. Research on fluorescent nanoparticles mainly concentrates on semiconductor particles, which are usually referred to as quantum dots. Among these, CdSe particles and ZnS particles have stronger fluorescent intensity. In spite of their broaden applications, quantum dots frequently still have some problems which are related to the intrinsic blinking of their luminescence and to toxicity issues that limit their applications in the health sciences [90]. Silver is expected to have lower toxicity and can be readily prepared reproducibly and with excellent solution stability. At the same time, Ag is readily detectable in the visible spectral region [91]. Because silver has the abovementioned advantages, the preparation of highly fluorescent silver nanoparticles is needed. Highly fluorescent silver nanoparticles were prepared by Maretti et al. [92] with a facile photochemical method, which can yield these materials with excellent long-term stability in just a few minutes. The method is used photogenerated ketyl radicals which can reduce Ag^+^ from silver trifluoroacetate in the presence of amines. The conclusion they obtained is that the luminescence arises from particle-supported small metal clusters (predominantly Ag_2_). Typically, silver nanoparticles show a distinct plasma band which has been between 390 and 420 nm in their past work. Due to the presence of small silver clusters, the study of the absorption band obtained was closer to 450 nm. Figure 15 shows the UV-vis absorption spectra of silver nanoparticles. Figure 16 shows the absorption (red), emission (green), and excitation (blue) spectra of Ag particles after 4 min of irradiation in tetrahydrofuran (THF) under the conditions of Fig. 15 and resuspension in toluene. From Fig. 16, we can draw the conclusion that the silver nanoparticles can emit green light. This property can be used for fluorescence diagnosis in biomedical field [93].

In order to distinguish these ultra-small particles, these nanoparticles which are smaller than 2 nm are usually called nanoclusters. In this size regime, metal nanoclusters become molecular species and size-dependent strong fluorescent emission can often be observed upon photoexcitation in the UV-visible range [94]. In particular, Ag nanoclusters, which show higher fluorescent intensity than Au nanoclusters in solutions, received considerable attention in the past few years owing to their great promise in a wide range of applications [95]. Fluorescent Ag nanoclusters were found to have wide applications in bio-imaging [96], chemical sensing [97, 98], fluorescence labeling [99], and single-molecule microscopy [100].

### Properties and Applications of AgNPs on Catalysis

**Fig. 17:**
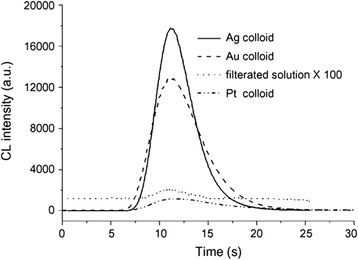
Effect of Ag colloid (solid line), 38 nm Au colloid (dashed line), Pt colloid (dash-dot-dot line), and filtrated solution of precipitated Ag colloid (dotted line) on luminol–H_2_O_2_ CL. The blank (filtrated solution of precipitated Ag colloid) signal was amplified by 100 times. Conditions: luminol, 1 × 10–4 mol/L; H_2_O_2_, 0.15 mol/L; pH 9.32 carbonate buffer for Ag, pH 12.0 NaOH for Au, pH 10.3 carbonate buffer for Pt [102]

Since the addition of silver nanoparticles into reaction, the catalytic performance of the reaction has been significantly improved. Thus, nanocatalysis of silver nanoparticles has been a rapid growing research area which involves the use of nanoparticles as catalysts. As we all know, metals such as Ag, Au, Pt, and other metal ions can catalyze the decomposition of H_2_O_2_ to oxygen [101]. Guo et al. found that when the AgNP colloid was added into the solution of luminol-H_2_O_2_, the chemiluminescence (CL) emission from the luminol–H_2_O_2_ system could be greatly enhanced. AgNPs exhibited a better catalytic performance of CL than gold and platinum nanoparticles. The AgNPs-enhanced CL was ascribed to that AgNPs could catalyze the decomposition of H_2_O_2_ to produce some reactive intermediates such as hydroxyl radical and superoxide anion. Figure 17 shows the effect of Ag colloid, Au colloid, Pt colloid, and filtrated solution of precipitated Ag colloid on luminol–H_2_O_2_ CL [102].

**Fig. 18:**
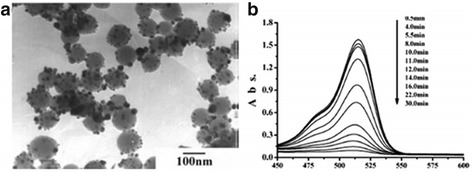
**a** Silver nanoparticles immobilized on silica spheres are illustrated. **b** The absorbance spectrum of the dyes decreases as the dyes are reduced by sodium borohydride. This process is catalyzed by silver nanoparticles. The arrow marks the increase of reaction time [104]

Silver is the most popular catalyst when it has interaction with oxygen, water, carbon dioxide, ethylene, and methanol [103]. From the study that the catalytic properties of silver nanoparticles have accordingly changed can be realized. Jiang et al. [104] enhanced the catalytic properties of Ag by combining silver nanoparticles with silica spheres, and they also applied it to the detection of dye reduction. The technique to support silver particles on silica spheres effectively avoids flocculation of nano-sized colloidal metal particles during a catalytic process in the solution, which allows one to carry out the successful catalytic reduction of dyes. Figure 18 shows how the absorbance spectrum of the dyes decreases when the dyes are reduced.

In addition, the catalytic properties of silver also have important applications in other areas, for example, wet-spun fibers [105].

### Properties and Applications of AgNPs on Surface Plasmon Resonance

In 1902, Wood found the SPR phenomenon for the first time in an optical experiment and made a brief record about that, but until in 1941, a scientist named Fano explained the phenomenon of SPR. Over the next 30 years, the theory about SPR has not been further explored nor has it been put into practical application. In 1971, Kretschmann put forward prism coupling structure that settled the foundation for the structure of SPR sensor, and SPR theory started to be widely achieved for experiments. On this basis, the surface plasma resonance effect of silver nanoparticles was explored deeply. The most successful part of the applications of plasmonic structures was in the detection of molecules. This technique has been commercialized for propagating surface plasmons (PSPs) on continuous metal films. The films are chemically functionalized to selective bind target molecules like DNA strands or proteins. Upon binding the target molecule, the dielectric environment is altered around the surface of the metal film. Consequently, binding can be monitored by measuring the change in coupling geometry (i.e., the angle) between the metal film and the excitation source needed to generate PSPs [106, 107]. This technique plays a key role, and a number of commercially available instruments are widely used today in the biological sciences [108].

**Fig. 19:**
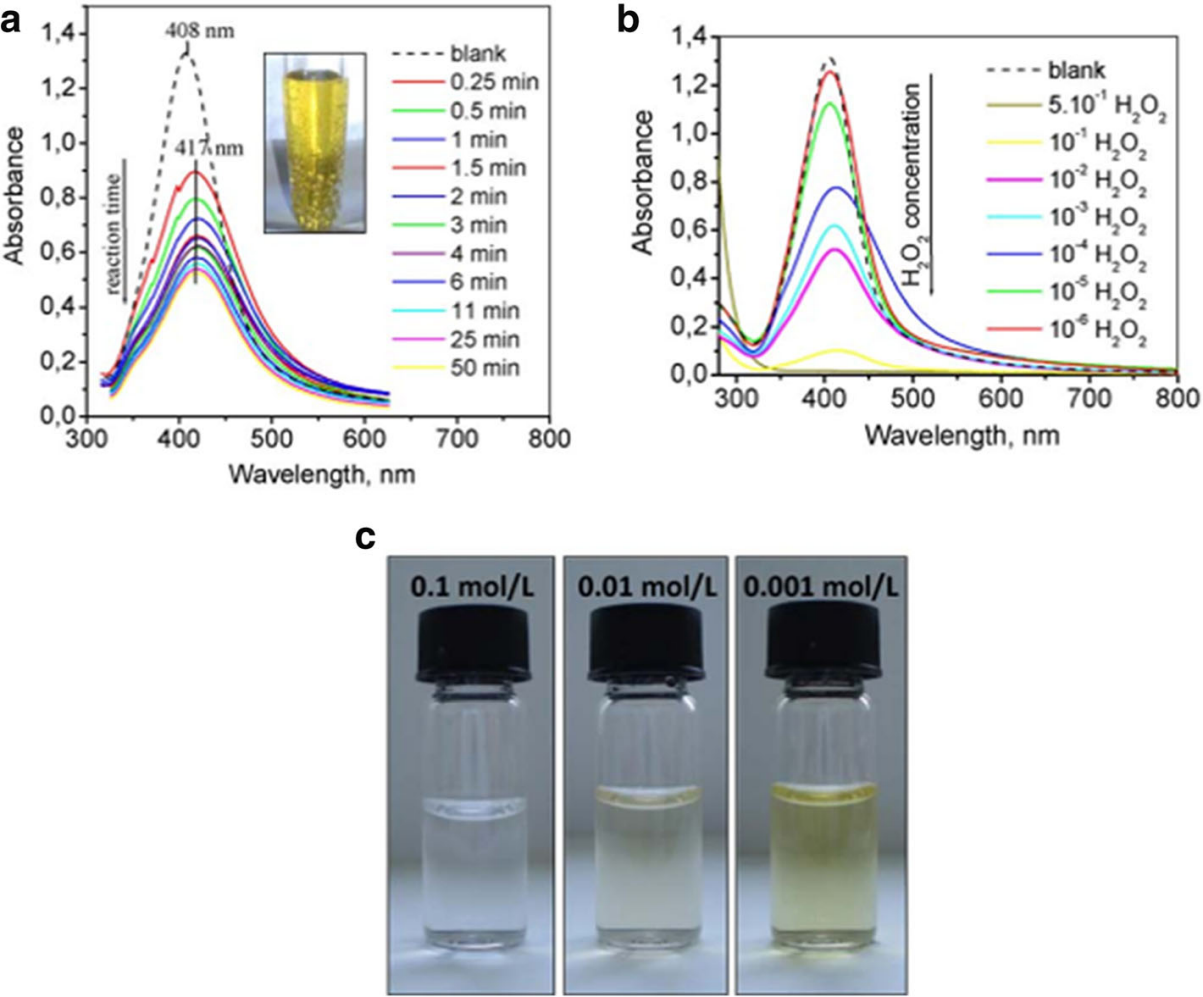
**a** Change of the LSPR absorbance strength with time due to the introduction of 10–3 mol/L H_2_O_2_ solution in the as-synthesized Ag-NPs solution at a volume ratio 1:1.5; the inset shows the bubbles from H_2_O_2_ decomposition generated by the catalytic reaction between hydrogen peroxide and starch-stabilized Ag-NPs. **b** UV-vis absorption spectra recorded 15 min after the introduction of hydrogen peroxide solution with different concentrations in the solution of Ag-NPs at a volume ratio 1:1.5. **c** relevant photographs of Ag-NPs dispersions 60 min after the introduction of hydrogen peroxide with different concentrations [109]

Recently, the combination of silver nanoparticles with other materials to improve their surface plasmon resonance performance is another way of development. The nanosilver particles were bonded with starch by Vasileva et al. [109], and the materials were applied as a surface plasmon resonance-based sensor of hydrogen peroxide. Figure 19 shows the change of hydrogen peroxide decomposition.

SPR has a wide range of applications in other fields such as life science, medical testing, drug screening, food testing, environmental monitoring, and forensic identification.

The SPR technology becomes an indispensable part in the field of biological chemistry, food, and drug monitoring. The applications of SPR biosensors will be more diversified. And especially its emerging application in small molecule detection and lipid field will make it play an increasingly important role in the film and biology. In recent years, its development is particularly rapid. With the continuous improvement of SPR instruments and the continuous enhancement of biological membrane construction capability, SPR biosensor has a bright future.

### Applications of AgNPs on Nanosensors

Due to the great research prospect of silver nanoparticles in nanosensors, many researchers have devoted to study it [110, 111]. So, we pick three representative examples to write in detail. Among them Zhu et al. [110] fabricated rhombic silver nanoparticles for biosensing. The rhombic silver nanoparticles were prepared by follow method. The mixed solution (polystyrene nanospheres and glass nanospheres with fluorocarbon surfactant) was coated onto the glass substrate to form a deposition mask, and then followed by hydrofluoric acid etching to remove the glass nanospheres. After that, the Ag metal thin film was deposited through the nanosphere masks using thermal evaporation or electron beam evaporation. After removal of the polystyrene nanospheres by sonication in absolute ethanol for 3 min, well-ordered rhombic AgNPs array was finally obtained on the substrates. The rhombic AgNPs array was single particle dimension of 140 nm in-plane width and 47 nm out-of-plane height. To prepare the biosensing, the Ag nanorhombuses are firstly functionalized using the self-assembly monolayer technique. Then assisting with 1-ethyl-3-[3-dimethylaminopropyl] carbodiimide hydrochloride, we covalently attached biotin to the carboxylate groups. The advantage of this biosensor is that the rhombic AgNPs array-based sensor with more hot spots has higher sensitivity than that of the traditional Ag triangular nanoparticles-based sensor. A detection of high sensitivity of the bio-molecule in lower concentration has been realized by means of the LSPR-based nanobiosensor. This type of biosensor will have potential applications in many fields such as medical science and biological technology. Meanwhile, M. Ghiaci et al. [111] utilized silver nanoparticles compounds as new electrochemical sensors for glucose detection. These electrochemical sensors were prepared based on synthesizing of two amine compounds bounded to silica support. The size of used AgNPs is 10 nm. The electrochemical sensor prepared by this method has a lower limit of glucose detection than other electrochemical sensors. This type of nanosensors will be more conducive to diabetes detection and treatment. Silver nanoscale sensors can also be used for environmental detection such as Li et al. [112] synthesized aza-crown ether (ACE)-modified silver nanoparticles as colorimetric sensors for Ba^2+^. What is more, colorimetric sensors merely need minimal instrumentation, achieve high sensitivity, and thus can make on-site detection even easier. The colorimetric sensors were synthesized by silver nanoparticles efficiently conjugated with CS_2_–ACE. ACE-modified AgNPs have good recognition of Ba^2+^, with the detection limit of 10^− 8^ mol/L.

In addition to the abovementioned, silver nanosensors also have other different applications that are worth us to explore.

### Other Applications

Ag nanomaterials also have many other applications in various fields, such as nanoscale detection [113] and solar cells.

Silver nanoparticle and its complex can be used for solar cells to enhance photoelectric conversion efficiency and photovoltaic performances [114–116].

Shen et al. [114] enhanced photovoltaic performances of polymer solar cells by incorporating Ag–SiO_2_ core–shell nanoparticles in the active layer. They creatively incorporated Ag–SiO_2_ core–shell nanoparticles (Ag–SiO_2_-NPs) into photo−/electro-active layers consisting of poly(3-hexylthiophene) (P3HT) and phenyl-C_61_-butyric acid methyl ester (PCBM) in polymer solar cells (PSCs). By this way, the photovoltaic performance of PSCs have largely been enhanced. The results demonstrate a 13.50% enhancement of short-circuit photocurrent density and a 15.11% enhancement of power conversion efficiency as the weight percent of doped Ag–SiO_2_-NPs is 1.5 wt% in the active layer of corresponding PSCs. In the later research, bare silver nanoplate (Ag-nPl) were spin-coated on indium tin oxide and silica capsulated Ag-NPs were incorporated to a PBDTTT-C-T:PC71BM active layer by Shen et al. [115]. As a result, the devices incorporated with Ag-nPl and Ag@SiO_2_-NPs showed great enhancements. With the dual effects of Ag-nPl and Ag@SiO_2_-NPs in devices, all wavelength sensitization in the visible range was realized; therefore, the power conversion efficiency of PSCs showed a great enhancement of 14.0 to 8.46%, with an increased short-circuit current density of 17.23 mA cm^− 2^. Importantly, the methodology of multiple shape combination of metallic nanoadditives improves the photovoltaic performance of PSCs very effectively compared to the single-shape method.

Thus, Ag is a promising material for the conversion of solar energy into electricity and good detection. In addition to the abovementioned, Ag also has many other applications, but it still needs people to further explore it.

## Conclusions

This work reviewed the development progress of Ag nanomaterials on synthesis methods and applications. Different shapes of Ag nanostructures had been synthesized such as cubic, rod-shaped, and sphere-shaped, Ag nanostructure obtained by chemical synthesis and microwave methods were successfully prepared. In addition, different size of AgNPs have been synthesized such as 1–10 nm, 10–100 nm, AgNPs obtained by chemical synthesis, laser ablation, and green synthesis. Meanwhile, it has been successfully applied to many fields, such as antibacterial, fluorescence, catalysis, SPR, and nanosensors, and it is expected to use in other fields. In fact, there are still limitations for their practical applications in photoelectric and medical fields because it often requires complex preparation process, and the yield is very low. In most cases, AgNPs are easy to agglomerate, which will greatly reduce its optical properties. Therefore, it is necessary to utilize surface active agent to achieve a good effect. Although, there are so many challenges, the advances in nanoscience and nanotechnology of silver still promise a better future for many kinds of industries. In conclusion, the future research of silver nanoparticles should be directed towards biosynthetic, size controllable, and uniform shape preparation. And the future application of AgNPs-based will be utilized in new energy battery or wearable intelligent equipment by its excellent localized surface plasmon resonance effect and antibacterial activity. In addition, AgNPs-based materials can be further utilized for applications in nanodevices by self-assembly and molecular molding technology.

## Abbreviations

ACE: Aza-crown ether
Ag-nPl: Silver nanoplate
AgNPs: Ag nanoparticles
CL: Chemiluminescence
CTAB: Cetyltrimethyl ammonium bromide
DBSA: Dodecyl benzene sulfonic acid
DLS: Dynamic light scattering
EDS: Energy dispersive spectroscopy
fcc: Face-centered cubic
HRTEM: High-resolution transmission electron microscopy
LSPR: Localized surface plasmon resonance
MTCC: The name of bacteria
MTPs: Multiply twinned particles
P3HT: Poly(3-hexylthiophene)
PAA: Poly(acrylic) acid
PBDTTT-C-T: Poly[4,8-bis((2-ethylhexyl)thiophen-5-yl)-benzo[1,2-b:4,5-b’]dithiophene-2,6-diyl]-alt-[2-(20-ethylhexanoyl)-thieno[3,4-b]thiophene-4,6-diyl]}
PC71BM: Fullerene derivatives acceptor material C71-butyric acid methyl ester
PCBM: Phenyl-C_61_-butyric acid methyl ester
PDDA: Poly(dimethyldiallylammonium chloride)
PEG: Polyethylene glycol
PLLA: Poly(L-lactide)
PSCs: Polymer solar cells
PSPs: Propagating surface plasmons
PVA: Poly-vinyl alcohol
PVP: Poly-vinyl pyrrolidone
SDA: Sabro dextrose agar
SDS: Sodium dodecyl sulfate
SEM: Scanning electron microscope
SERS: Surface-enhanced Raman scattering
SPR: Surface plasmon resonance
TEM: Transmission electron microscope
THF: Tetrahydrofuran
UV-vis: Ultraviolet-visible
XRD: X-ray diffraction
